# The Impact of Bacterial–Fungal Interactions on Childhood Caries Pathogenesis

**DOI:** 10.3390/pathogens14101033

**Published:** 2025-10-11

**Authors:** Shiyan Huang, Haojie Wang, Jing Tian, Man Qin, Ruixiang Gao, Bingqian Zhao, Jingyan Wang, Huajun Wu, He Xu

**Affiliations:** 1 Department of Pediatric Dentistry, Peking University School and Hospital of Stomatology & National Center for Stomatology & National Clinical Research Center for Oral Diseases & National Engineering Research Center of Oral Biomaterials and Digital Medical Devices & Beijing Key Laboratory of Digital Stomatology, Beijing 100081, China; huangsy0404@163.com (S.H.); jtian@pkuss.bjmu.edu.cn (J.T.); qin-man@foxmail.com (M.Q.); zhaobingqian@pku.edu.cn (B.Z.); jenkins_1211@163.com (J.W.); 2 Second Clinical Division, Peking University School and Hospital of Stomatology, Beijing 100101, China; 3 Key Laboratory of Carcinogenesis and Translational Research (Ministry of Education), Peking University Cancer Hospital and Institute, Beijing 100142, China; hjwang@genetics.ac.cn (H.W.); gaorx24@163.com (R.G.); hjwu@pku.edu.cn (H.W.); 4 Center for Precision Medicine Multi-Omics Research, Institute of Advanced Clinical Medicine, Peking University, Beijing 100191, China

**Keywords:** childhood caries, saliva, microbiota, mycobiota, bacteria, fungus

## Abstract

Caries is the most prevalent chronic disease affecting oral health in preschool children. In this 12-month prospective cohort study of 3–4-year-olds, we investigated the community-level bacterial–fungal interkingdom interactome and its role in cariogenic microenvironments, using 16S rRNA gene (bacterial) sequencing and ITS2 gene (fungal) sequencing of unstimulated saliva. Longitudinal analysis identified 19 key bacterial and fungal species that were associated with both caries progression and clinical features. Salivary bacteria *Desulfovibrio*, *Bacteroides heparinolyticus*, *Alloprevotella*, *Anaerobiospirillum*, and fungus *Candida tropicalis* not only showed altered abundances during caries development but also correlated with severity of caries, establishing diagnostic microbial signatures for caries prediction. The salivary mycobiome exhibited highly active and complex intra-network interactions in the caries-active state, suggesting that fungal networks may drive the broader community-wide microbiota interaction network in the caries state. Metabolic profiling further revealed distinct pathway shifts before and after caries onset. The findings demonstrate that caries progression follows ecological succession governed by cross-domain interactions. This study highlighted the fungal network’s important role in driving dysbiosis, advancing the current understanding of early childhood caries beyond bacterial-centric models, and also highlighted fungi not only as modulators but as active contributors to cariogenesis, which could guide future antimicrobial strategies.

## 1. Introduction

Dental caries represents the predominant oral disease in children globally, not only exerting adverse effects on their physical and mental well-being but also imposing a substantial economic burden on society [1,2,3]. Data from the 2018 National Oral Health Epidemiological Survey of China indicated that the caries prevalence rate among 3-year-old children reached 50.8%, while that among 5-year-old children was as high as 71.9% [1]. From the perspective of the pathogenesis of childhood dental caries, accurate prediction of children’s caries risk would not only contribute to reducing the incidence of dental caries but would also help to alleviate the significant economic burden on families and society.

Dental caries is a multifactorial biomineral disease of dental hard tissue arising from the dynamic interplay between cariogenic microbiota and host susceptibility within the salivary microenvironment. Saliva, governing the homeostasis of the oral ecosystem, serves as a critical experimental matrix for elucidating cariogenic mechanisms [4]. Furthermore, saliva-based diagnostics, characterized by non-invasiveness, technical feasibility, and high specificity, render it particularly amenable for investigations involving pediatric populations with developing dentition.

Previous investigations have established that the dynamic shift in the salivary micro-biome during caries progression exhibits discernible spatiotemporal patterns. These alterations are characterized by reduced microbial diversity and distinct inter-microbial interactions compared with caries-free children [5]. These findings suggest that the features of microbial interactions might serve as discriminators for caries risk stratification in pediatric populations [6]. However, the oral ecosystem harbors diverse fungal communities alongside bacteria. Despite their comparatively low abundance, fungi significantly influence host health and disease states [7]. Currently, more than 100 species of fungi have been detected in oral cavities. Cross-sectional analyses demonstrate characteristic spatiotemporal distribution patterns of fungi in children associated with caries onset [8,9]. Nevertheless, the literature predominantly examines bacterial or fungal communities in isolation, leaving the functional significance of cross-kingdom microbial interactions in the cariogenic process inadequately characterized.

In addition, our prior work on caries-associated metabolic characteristics of oral microbiota revealed functional shifts in bacterial carbohydrate metabolism during cariogenesis [10]. Cross-sectional evidence further delineated discriminative metabolomic signatures in plaque and saliva between caries-active and caries-free states, indicating substantial microbial metabolic reprogramming in caries pathogenesis [11,12]. Nevertheless, no integrated analysis to date has concurrently examined ecological networks, structural shifts, and functional adaptations to holistically elucidate the role of the oral microbiome dynamics in the cariogenic trajectory.

Against the aforementioned research background, this study recruited 3-to-4-year-old children with healthy primary dentitions to conduct a 12-month prospective cohort study. Utilizing high-throughput sequencing analysis of salivary microbiota, we aimed to elucidate the compositional profiles of oral bacteria and fungi, as well as the intra- and inter-kingdom microbial interactions along with the cariogenesis. Furthermore, we intended to identify possible microbial biomarkers for caries prediction. This study is expected to further elucidate the mechanism underlying the occurrence of childhood dental caries at the microbiota level to shed light on the prediction of childhood caries occurrence, as well as the development of anti-caries agents.

## 2. Materials and Methods

### 2.1. Ethical Statement

The study design, protocol, and informed consent procedures were approved by the Ethics Committee of Peking University Hospital of Stomatology (PKUSSIRB-202171207). Written informed consent was obtained from parents or guardians of all the participants at enrollment.

### 2.2. Prospective Cohort Management

**Figure 1:**
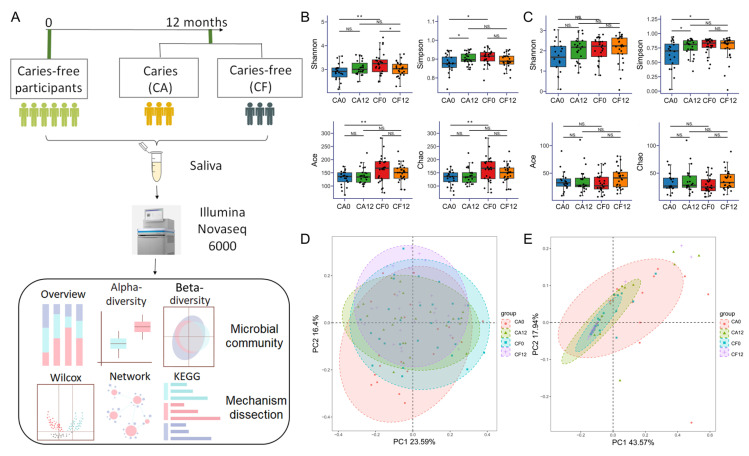
Bacterial community and fungal community diversity variation during the development of childhood caries. (**A**) Flowchart of the study. (**B**) Alpha diversity analysis of bacterial communities in each group. *: *p* < 0.05; **: *p* < 0.01; NS.: *p* > 0.05. (**C**) Alpha diversity analysis of fungal communities in each group. *: *p* < 0.05; NS.: *p* > 0.05. (**D**) PCoA plot based on Bray–Curtis dissimilarity matrix showed no significant difference in salivary bacterial community composition among the four groups. PC1 accounts for 23.59% of the variance, and PC2 accounts for 16.4% of the variance. *p* < 0.05, R^2^ = 0.054. (**E**) PCoA plot based on Bray–Curtis dissimilarity matrix showed no significant difference in salivary fungal community composition among the four groups. PC1 accounts for 43.57% of the variance, and PC2 accounts for 17.94% of the variance. *p* < 0.05, R^2^ = 0.078.

A total of 120 3-to-4-year-old healthy children from 5 kindergartens in Haidian District, Beijing, underwent routine oral examination during September 2022 to March 2023. Among them, 64 caries-free and healthy children were recruited and followed-up for 12 months. Oral examination and saliva sampling were carried out at both enrollment and the end of the observation. Based on the different states of being caries-free and caries-active at follow-up, the participants were divided into the caries-free group (CF) and the caries-active group (CA) (Figure 1A). There were four groups: (1) CA0 group—CA children at baseline, (2) CA12 group—children detected with active caries at 12 months, (3) CF0 group—CF children at baseline, and (4) CF12 group—children who remained caries-free at 12 months. 

All the children shared the same food menu at kindergarten and maintained consistent oral hygiene habits during the daytime. Parents were given instructions of standardized oral health guidance on brushing and flossing before recruitment.

Inclusion criteria: (1) healthy 3–to-4-year-old children with complete primary dentition; (2) caries-free, no white spot lesions, no dental developmental diseases (e.g., enamel hypoplasia), and no salivary gland disorders; (3) systemically healthy with no history of systemic diseases or use of medications; normal maternal pregnancy history; (4) no systemic antibiotics or fluoride application within 1 month prior to sampling; (5) no infectious diseases (e.g., upper respiratory tract infection) at the time of sampling. Exclusion criteria: (1) failure to cooperate; (2) wearing an orthodontic appliance during the observation.

### 2.3. Oral Examination and Saliva Collection

Oral examination of all the participants was performed by one experienced pediatric dentist under natural light. Examination consistency was ensured by conferring with two other attending dentists prior to the initiation of the study. The κ value for intra-examiner agreement in the diagnosis of caries was 0.88, indicating **“** almost perfect agreement” between the examiners. Caries status and the decayed, missing, and filled teeth (dmft) index were scored according to the World Health Organization caries diagnostic criteria (5th edition, 2013): caries lesions involving the dentin, cavitated lesions, or softened areas in the enamel on occlusal or smooth surfaces were considered to be caries [13]. Evaluation of the oral hygiene status was conducted through plaque index (PLI) assessment using the Turesky-modified Quigley–Hein plaque index. The scoring criteria were as follows: 0 = no plaque; 1 = isolated flecks of plaque at the gingival margin; 2 = thin plaque ribbon ≤ 1 mm at the gingival margin; 3 = plaque ribbon > 1 mm but occupying < 1/3 of the tooth surface; 4 = plaque covering 1/3–2/3 of the tooth surface; 5 = plaque covering > 2/3 of the tooth surface. No radiographs were taken [14].

All the participants were instructed to refrain from eating, brushing teeth, drinking water, exercising, or chewing gum for one hour, and to rinse their mouths thoroughly with water approximately 10 min before sampling. Unstimulated saliva was collected using a draining method for five minutes, which was conducted during 9:00 to 10:00 a.m. [15]. Essentially, the children were instructed to tilt their heads slightly forward, position their tongues against their palates, and let their saliva flow naturally into a sterilized 15 mL centrifuge tube. Samples were immediately put on ice and transferred to the laboratory within 2 h. Saliva was centrifuged at 500× *g* for 10 min at 4 °C. The supernatant was collected and stored at −80 °C until further analysis.

### 2.4. DNA Extraction, Amplification, and Illumina Novaseq Sequencing

Bacterial and fungal DNA were extracted from saliva samples using the QIAamp^®^ DNA Micro Kit (QIAGEN, Hilden, Germany) [16]. The sample quality was verified by using nanodrop spectrophotometry. PCR amplification was performed using 2 × Taq Plus Master Mix (Takara, Shiga, Japan) for the 16S rRNA gene V3–V4 region (forward primers: 5′-CCTACGGGNGGCWGCAG-3′, reverse primers: 5′-GACTACHVGGGTATCTAATCC-3′) and fungal ITS2 region (forward primers fITS7F: 5′-GTGARTCATCGAATCTTTG-3′, reverse primers ITS4R: 5′-TCCTCCGCTTATTGATATGC-3′) [17]. The degenerate bases follow IUPAC nomenclature: W = A/T, H = A/C/T, V = A/C/G, R = A/G. To clarify, these labels (W, H, V, R) specifically indicate the use of a mixture of primers that includes all relevant nucleotide variations. The PCR products were approximately 200–300 bp in size.

The PCR product was extracted from 2% agarose gel and purified using the AxyPrep DNA Gel Extraction Kit (Axygen Biosciences, Union City, CA, USA) according to the instructions of the manufacturer and quantified using a Quantus™ Fluorometer (Promega, Madison, WI, USA). Purified amplicons were pooled in equimolar and paired-end sequences on an Illumina MiSeq PE300 platform/NovaSeq PE250 platform (Illumina, San Diego, CA, USA) according to the standard protocols by LianChuan Technology Co., Ltd. (Hangzhou, China).

The raw sequence data reported in this paper have been deposited in the Genome Sequence Archive (Genomics, Proteomics & Bioinformatics 2021) in the National Genomics Data Center (Nucleic Acids Res 2025), China National Center for Bioinformation/Beijing Institute of Genomics, Chinese Academy of Sciences (GSA: CRA026916; CRA027046), which are publicly accessible at https://ngdc.cncb.ac.cn/gsa.

### 2.5. Sequencing Data Quality Control

Raw 16S and ITS1 rRNA gene sequencing reads were demultiplexed and quality-filtered using Fastq, then merged using FLASH with the following criteria [18,19]: (I) a 50 bp sliding window with an average quality score ≥ 20 was applied, and sequences shorter than 120 bp were discarded; (II) an overlap of at least 10 bp with a maximum mismatch rate of 0.1 was required for sequence merging, generating final Fasta sequences.

### 2.6. Bioinformatics and Data Analysis

Using the QiimeII pipeline (confidence threshold = 0.7), amplicon sequence variants (ASVs) were annotated by aligning representative sequences against the Human Oral Microbiome Database (HOMD, v15.2) for bacteria and the UNITE database (v9.0) for fungi to characterize taxonomic composition and distribution [20].

Analyses were performed using R packages V 4.5.1 [21]. Alpha diversity indices (Chao1, Shannon, Simpson, and ACE) were calculated using the vegan package in R (similarity threshold = 0.97). Group differences were compared using the Kruskal–Wallis H test followed by Tukey’s test (or Student’s *t*-test, where applicable). Adjusted *p*-values were obtained via the Benjamini–Hochberg method. Bray–Curtis distance matrices were computed with vegan for nonparametric multivariate analysis of variance (PERMANOVA) to assess community compositional similarities/differences (*p* < 0.05 indicated significance). Principal coordinate analysis (PCoA) was performed to visualize beta diversity patterns. Relative abundances of microorganisms (derived from absolute abundance data filtered by QiimeII) were analyzed using the Wilcoxon rank-sum test (Wilcox analysis). Microbes with a fold change > 1.5 or <2/3 and *p* < 0.1 were identified as differentially abundant between groups. Clinical features (dmft and PLI indices) in the CF12 and CA12 groups were correlated with microbial abundances using Spearman’s rank correlation via the Hmisc package in R. Random forest classifiers (via the randomForest package in R) were built to distinguish the CA and CF groups, screening for indicator microbes predictive of caries. The out-of-bag error (OOB) was calculated to evaluate model performance, and the mean decrease in Gini coefficient (Mean Decrease Gini) was used to rank the importance of microbial features. Spearman correlation matrices were computed with Hmisc, and microbial co-occurrence networks were visualized using the igraph package to characterize intra-kingdom (bacterial/fungal) and interkingdom (bacterial–fungal) interaction patterns. Network topological properties (e.g., connectivity, modularity) were compared across groups to analyze interaction network characteristics. Microbial metabolic functions were predicted using PICRUSt2 based on 16S rRNA ASV sequences. Predicted functional profiles were annotated against the Kyoto Encyclopedia of Genes and Genomes (KEGG) pathway database. Functional abundance distributions were visualized with ggplot2 in R, and differentially enriched metabolic pathways between groups were identified via linear discriminant analysis effect size (LEfSe) using the microeco package in R.

## 3. Results

### 3.1. Participants and General Sequencing Information

Sixty-four caries-free children were recruited at baseline of the prospective cohort. After 12 months of follow-up, six participants dropped out due to various reasons, while another six participants only underwent oral examination without saliva collection at the end of the observation. Ultimately, 52 participants with complete longitudinal datasets were included. They were divided into 28 caries-free (CF group) and 24 caries-active (CA group) participants based on their caries status at follow-up (Figure 1A). No significant differences were observed in gender or age (months) between the two groups (Tables S1 and S2). At the 12-month endpoint, children in the CA group (CA12) exhibited no restored or exfoliated teeth due to caries. Therefore, the decayed tooth (dt) index was applied for subsequent statistical analysis. The mean dt value of the CA12 group was 3.54 ± 1.86.

### 3.2. Variation in the Salivary Bacterial Community and Fungal Community During Caries Development

In this prospective cohort, both salivary bacterial and fungal communities exhibited distinct compositional and diversity changes during caries onset. Longitudinal comparisons in the CA group revealed intra-host microbial dynamics tracking cariogenic progression, whereas fluctuations in the CF group reflected physiological variations within a stable ecological state.

Specifically, the alpha diversity of the salivary bacterial community, which is the measure of species richness and evenness within a single ecological community, was significantly lower in the CA0 group compared with the CF0 group (*p* < 0.05; Figure 1B). No significant differences were observed in the alpha diversity of salivary fungi among the four groups (*p* > 0.05, Figure 1C) This indicates that salivary bacterial diversity differed between caries-susceptible children and healthy children even prior to the clinically caries occurrence, which was aligned with previous longitudinal study findings [22]. No significant difference in microbial beta diversity was observed (*p* > 0.05) (Figure 1D,E), suggesting that early compositional shifts may primarily occur at the ASV level rather than through major community restructuring.

#### 3.2.1. Shift in Salivary Bacterial Community Composition During Caries Development

High-throughput sequencing of bacterial 16S rRNA genes yielded 11,628 amplicon sequence variants (ASVs) after clustering, aligning with an average of 3168 ASVs per sample. Annotation against the HOMD database identified 22 phyla, 36 classes, 60 orders, 115 families, 345 genera, and 286 species (Figure S1A).

**Figure 2:**
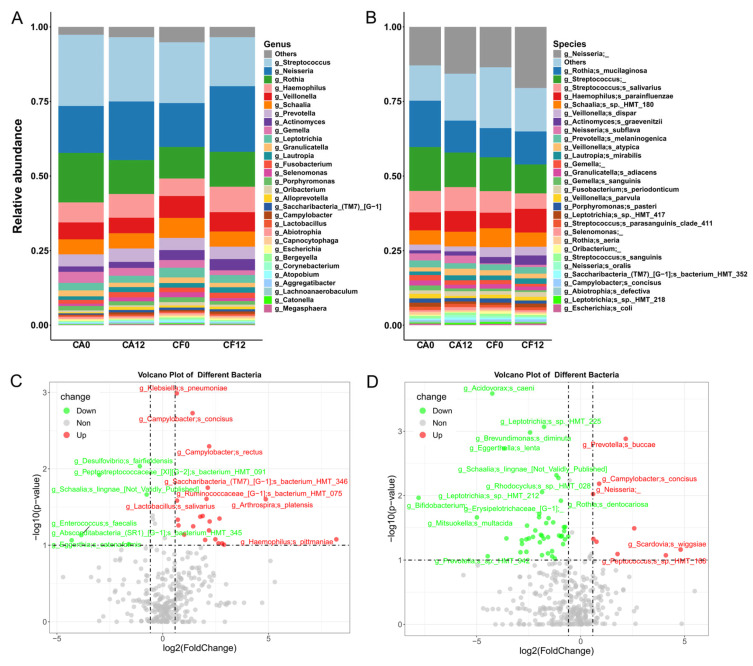
Alterations in salivary bacterial community composition associated with caries occurrence in children. (**A**) Taxonomic distribution of the relative abundances of reads of major bacterial genera. (**B**) Taxonomic distribution of the relative abundances of reads of major bacterial species. (**C**) Comparison of relative abundances in bacteria species between the CA0 and CA12 groups. (**D**) Comparison of relative abundances in bacteria species between the CF0 and CF12 groups. Using the Wilcoxon rank-sum test, *p* < 0.05 was considered to reflect a significant difference. Red indicates species with a significant increase in abundance from baseline to follow-up, green indicates species with a significant decrease in abundance, and gray indicates species with no statistical difference between the two groups. The dotted line in the volcano plot represents the threshold, log2(FoldChange) = 1.5.

At the genus level, the top six most abundant genera in all four subgroups (CA0, CA12, CF0, CF12) were *Streptococcus*, *Neisseria*, *Rothia*, *Haemophilus*, *Veillonella*, and *Schaalia*, collectively accounting for >70% of the total bacterial community (Figure 2A). At the species level, the top six species across subgroups were *Neisseria* unspecified, *Rothia mucilaginosa*, *Streptococcus* unspecified, *Streptococcus salivarius*, *Haemophilus parainfluenzae*, and *Schaalia* sp.*_HMT_180*, comprising > 55% of the total community (Figure 2B, Table S3). However, relative abundances of these species varied significantly between groups. The significantly increased bacterial species along with caries development in the CA group included *Lactobacillus salivarius*, *Klebsiella pneumoniae*, *Campylobacter concisus*, *Campylobacter rectus*, *Haemophilus parainfluenzae*, and *Streptococcus anginosus*, which were consistent with trends reported in previous studies. Conversely, the significantly decreased bacterial species included *Brevundimonas diminuta*, *Rothia dentocariosas*, and *Bifidobacterium* (Figure 2C,D). No relative abundance difference was detected in these species in the CF group during the observation (Figure 2D).

#### 3.2.2. Shift in Salivary Fungal Composition During Caries Development

High-throughput sequencing of fungal ITS genes generated 2805 ASVs post-clustering, aligning to an average of 94 ASVs per sample. Annotation against the UNITE (v9.0) database identified 9 phyla, 29 classes, 73 orders, 165 families, 286 genera, and 543 species (Figure S1B).

**Figure 3:**
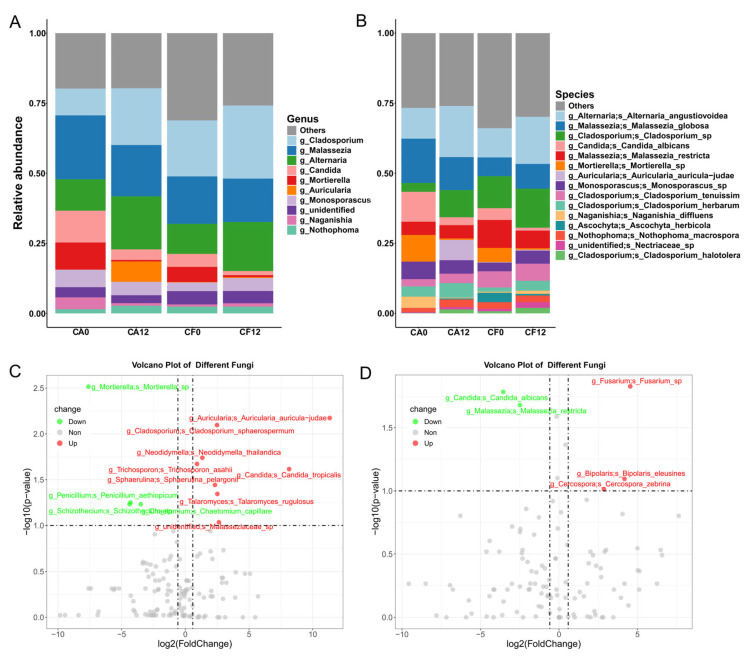
Alterations in salivary fungal community composition associated with caries occurrence in children. (**A**) Taxonomic distribution of the relative abundances of reads of major fungal genera. (**B**) Taxonomic distribution of the relative abundances of reads of major fungal species. (**C**) Comparison of relative abundances in fungal species between the CA0 and CA12 groups. (**D**) Comparison of relative abundances in fungal species between the CF0 and CF12 groups. Using the Wilcoxon rank-sum test, *p* < 0.05 was considered to reflect a significant difference. Red indicates species with a significant increase in abundance from baseline to follow-up, green indicates species with a significant decrease in abundance, and gray indicates species with no statistical difference between the two groups. The dotted line in the volcano plot represents the threshold, log2(FoldChange) = 1.5.

At the genus level, although the dominant fungal genera varied among the four subgroups, the top three most abundant genera remained *Cladosporium*, *Malassezia*, and *Alternaria*, which are commonly detected in nature and from human skin (Figure 3A). At the species level, the quantity of fungal species was much less than that of bacterial species. Major species included *Alternaria angustiovoidea*, *Malassezia globosa*, *Cladosporium* sp., *Candida albicans*, and *Malassezia restricta* (Figure 3B, Table S4). During caries development, increased fungi species included *Candida tropicalis*, *Sphaerulina pelargonii*, *Talaromyces rugulosus*, and *Cladosporium sphaerospermum*, while decreased fungi species included *Mortierella* sp., *Chaetomium capillare*, *Schizothecium* sp. and *Penicillium aethiopicum* (Figure 3C,D).

### 3.3. Salivary Bacterial and Fungal Species Associated with Caries Clinical Feature

**Figure 4:**
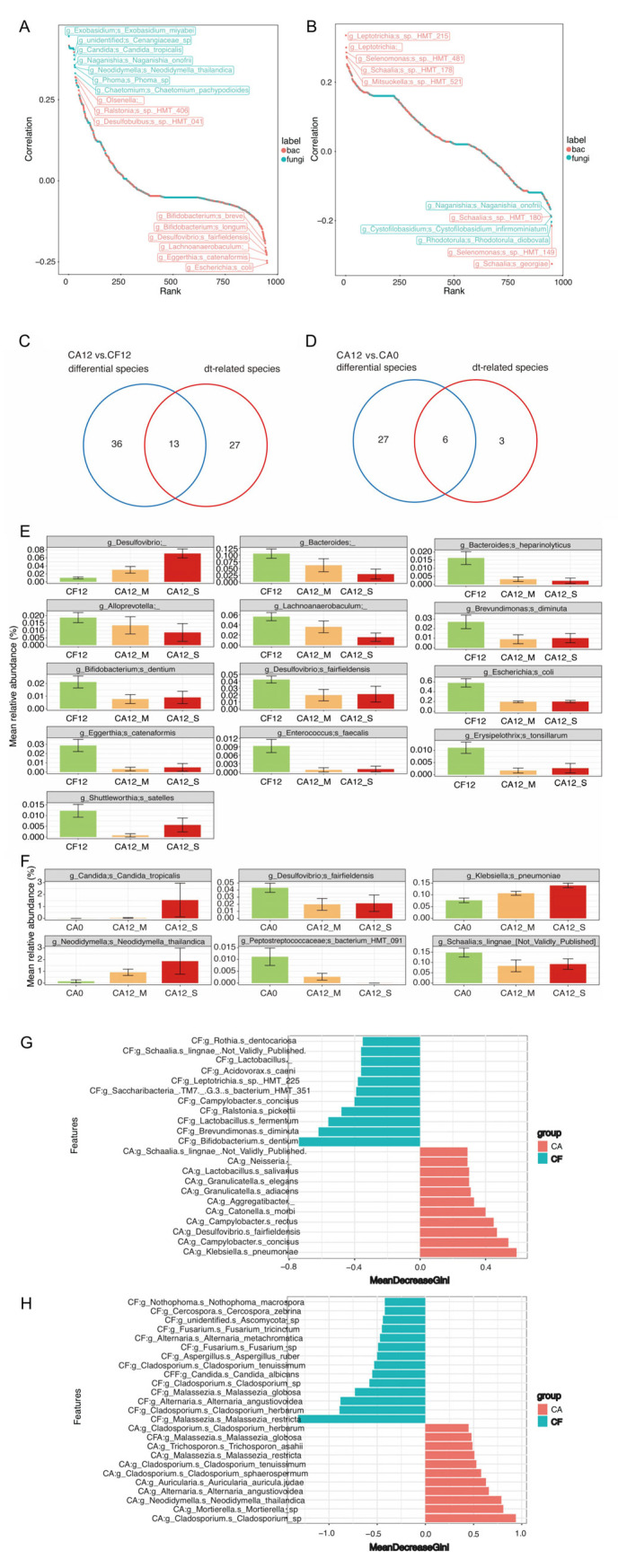
Clinical Feature associated and Caries-discriminatory Bacteria and Fungi. (**A**) Correlation between clinical characteristic dt value and bacterial/fungal species. (**B**) Correlation between clinical characteristic PLI index and bacterial/fungal species. Using Spearman’s rank correlation analysis, red indicates bacteria and blue indicates fungi. (**C**) Venn diagram screened the intersected bacterial and fungal species from both dt value (dental caries index)-related species and the significantly varied species between CA12 and CF12 groups. (**D**) Venn diagram screened the intersected bacterial and fungal species from both dt value (dental caries index)-related species and the significantly varied species between CA12 and CA0 groups. (**E**) Relative abundance distribution of the key microorganisms in the CF12 and CA12 groups. The abscissa represents sample grouping (CA12 is divided into two subgroups based on dt value: CA12_M subgroup: dt < 4, CA12_S subgroup: dt ≥ 4), and the ordinate represents relative abundance. (**F**) Relative abundance distribution of the key microorganisms in the CA0 and CA12 groups. The abscissa represents sample grouping (CA12 is divided into two subgroups based on dt value: CA12_M subgroup: dt < 4, CA12_S subgroup: dt ≥ 4), and the ordinate represents relative abundance. (**G**) Bacterial random forest model analysis for caries prediction. (**H**) Fungal random forest model analysis for caries prediction. Red indicates species that contribute to caries development, blue indicates species that contribute to caries-free maintenance.

The dmft index represents the level of caries severity in children of the CA group. In this study, Spearman correlation analysis identified the specific species that were associated positively with the dt index, including bacteria *Olsenella* and *Desulfovibrio* and fungi *Exobasidium miyabei* and *Candida tropicalis*. Meanwhile, negative correlations were observed with *Bifidobacterium breve*, *Bifidobacterium longum*, *Eggerthella lenta*, and *Escherichia coli* (Figure 4A).

The Plaque Index (PLI) can serve as a simplified representation of the overall oral hygiene status. Bacterial species that positively correlated with PLI included *Leptotrichia* sp.*_HMT_215* and *Selenomonas* sp.*_HMT_481*, whereas negatively correlated species included *Naganishia onofrii*, *Rhodotorula dibositava*, and *Selenomonas* sp.*_HMT_149* (Figure 4B). These species were primarily commensal oral bacteria, although *Selenomonas* has been reported to associate with caries development [23].

Furthermore, nineteen species were identified as intersection of both the significantly varied species and the dt index related species (Figure 4C,D). These species were considered as the key species that associated with caries progression. Among them, the relative abundance of *Desulfovibrio* sp. increased along with dt value, while species *Bacteroides* sp., *Bacteroides heparinolyticus*, *Alloprevotella* sp. and *Anaerobiospirillum* sp. exhibited the opposite trend. In addition, species *Brevundimonas diminuta*, *Bifidobacterium dentium*, *Desulfovibrio fairfieldensis*, *E. coli*, *Eggerthella lenta*, *Enterococcus faecalis*, *Erysipelothrix tonsillarum*, and *Shuttleworthia satelles* also showed lower abundances in the CA12 group than the CF12 group (Figure 4E). Meanwhile, relative abundance of *Candida tropicalis*, *Klebsiella pneumoniae* and *Neodidymella thailandica* increased along with caries development and severity, while *Desulfovibrio fairfieldensis*, *Peptostreptococcaceae_[XI][G−2] bacterium_HMT_091*, and *Schaalia lingnae* showed higher abundances in the CA0 group than the CA12 group (Figure 4F).

### 3.4. Caries-Discriminatory Bacteria and Fungi in Saliva

Random forest models were constructed to predict caries occurrence, based on the species-level bacterial and fungal data.

For predicting caries-free maintenance, the top five contributing bacterial species were *Bifidobacterium dentium*, *Brevundimonas diminuta*, *Lactobacillus fermentum*, *Ralstonia picketti*, and *Campylobacter concisus*, while the top five contributing fungus species were *Malassezia restricta*, *Cladosporium herbarum*, *Alternaria angustiovoidea*, *Malassezia globosa*, and *Cladosporium* sp., with the addition of *Candida albicans* ranking sixth (Figure 4G,H).

For predicting caries development, the top five bacterial species were *Klebsiella pneumoniae*, *Campylobacter concisus*, *Desulfovibrio fairfieldensis*, *Campylobacter rectus*, and Catonella morbi (Figure 4G). Among these, *K. pneumoniae*, *C. concisus*, and *C. rectus* exhibited increased relative abundances during caries progression. Accordingly, the top five contributing fungus species for predicting caries development were *Cladosporium* sp., *Mortierella* sp., *Neodidymella thailandica*, *Alternaria angustiovoidea*, and *Auricularia auricu-la-judae* (Figure 4H); of which *Mortierella* sp. showed increased relative abundance during caries development.

### 3.5. Network Analysis of the Intra-Kingdom and Interkingdom Interactions During Caries Development

**Figure 5:**
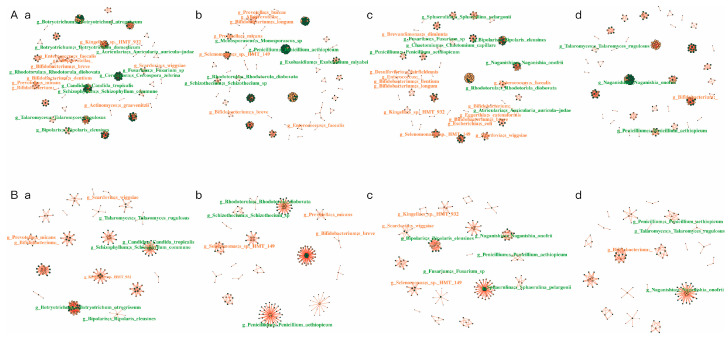
Interaction networks of salivary bacterial and fungal communities during caries development. Correlations with Spearman correlation coefficient |r| > 0.9 and *p* < 0.01 are shown. Red indicates bacterial species and green indicates fungi species. Edges indicate an association between species. (**A**) Overall interaction networks of the whole salivary bacterial community: a. CA0 group; b. CA12 group; c. CF0 group; d. CF12 group. (**B**) Interkingdom interaction networks of bacteria–fungi crosstalk: a. CA0 group; b. CA12 group; c. CF0 group; d. CF12 group.

**Figure 6:**
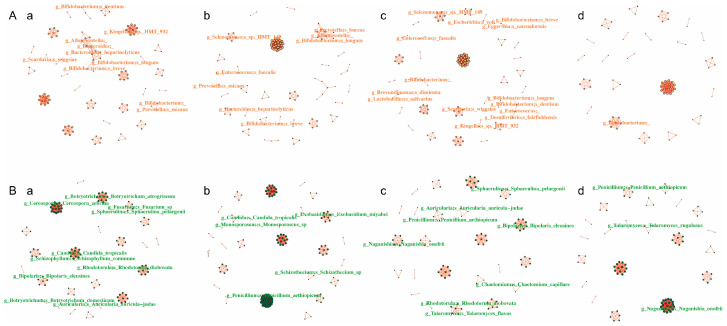
Intra-kingdom interaction networks of salivary bacterial and fungal communities during caries development. Correlations with Spearman correlation coefficient |r| > 0.9 and *p* < 0.01 are shown. Red indicates bacterial species and green indicates fungi species. Edges indicate an association between species. (**A**) Intra-kingdom interaction networks within salivary bacterial community: a. CA0 group; b. CA12 group; c. CF0 group; d. CF12 group. (**B**) Intra-kingdom interaction networks within salivary fungal community: a. CA0 group; b. CA12 group; c. CF0 group; d. CF12 group.

Network analysis was performed to explore both the intra-kingdom and interkingdom interactions of salivary bacterial community and fungal community during the caries development process (Table 1, Figure 5 and Figure 6). In the entire interaction network (encompassing all bacteria and fungi), the four groups possessed similar node numbers. However, the CA12 group demonstrated the highest number of edges and the greatest network connectivity. Within the cross-kingdom (bacteria to fungi) networks, the CA group (at both time points) exhibited a higher number of associated nodes and edges compared with the CF group. Within the bacterial community network, the CA0 group exhibited the highest number of edges and the highest average network connectivity, whereas the CA12 group had the lowest number of nodes and edges. Notably, within the mycobiota network, the CA12 group displayed the highest number of nodes, edges, and average network connectivity. It can be predicted that when caries is present, salivary fungi exist in a state characterized by highly active and complex interactions. Intraspecific interactions within the fungal community is likely the primary driver of the entire microbiome activity at the caries-active state, which is likely to contribute to the development of caries in preschool children.

### 3.6. Prediction of Metabolic Pathways of the Salivary Microbiome in Relation to Childhood Caries

**Figure 7:**
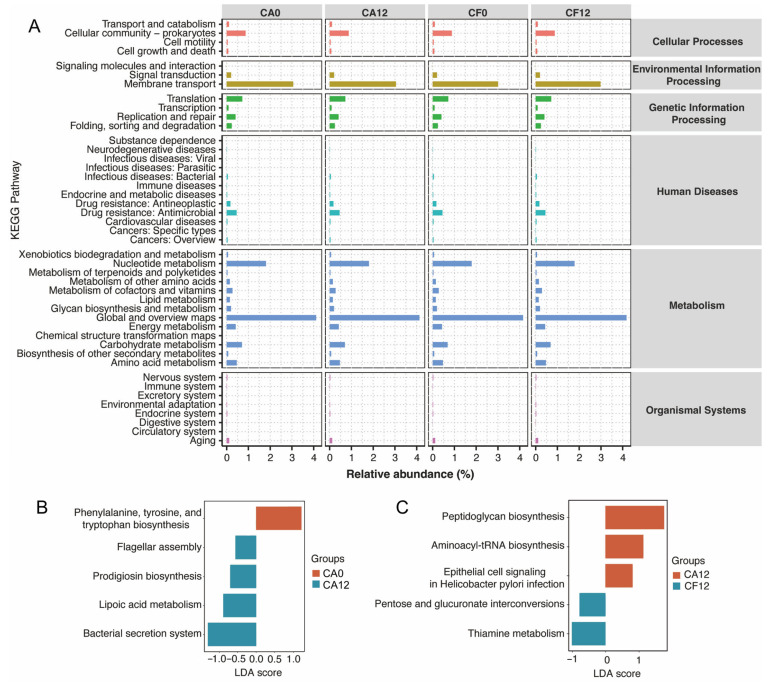
Variation in functional profiles of the salivary microbiome during childhood caries development. (**A**) Prediction of KEGG functional pathways of the salivary microbiome was performed using PICRUSt2 based on the 16S rRNA and ITS gene sequencing data. The right side shows the KEGG pathways at Level 1. The left side shows the KEGG pathways at Level 3. The plot shows the relative abundance of the KEGG pathways. (**B**) LEfSe analysis of different functional pathways between the CA0 and CA12 groups. Red indicates the CA0 group, while blue indicates the CA12 group. Only pathways with a significant LDA score (LDA score > 0.5, *p* < 0.05) are shown here. (**C**) LEfSe analysis of different functional pathways between the CA12 and CF12 groups. Red indicates the CA12 group, while blue indicates the CF12 group. Only pathways with a significant LDA score (LDA score > 0.5, *p* < 0.05) are shown here.

Through alignment with the KEGG database, enrichment analysis revealed that the potential functional pathways involved in the salivary microbiota were primarily enriched in Metabolism, Environmental Information Processing, Genetic Information Processing, and Cellular Processes; with the most active pathways including Prokaryotic Cell Community, Membrane Transport, Nucleotide Metabolism, Global and Overview Maps, Carbohydrate Metabolism, and Amino Acid Metabolism (Figure 7A).

In the CA group, prior to the detection of dental caries (CA0), the functional profile of the microbiota exhibited greater activity in the pathways of phenylalanine, tyrosine, and tryptophan biosynthesis; after caries occurrence (CA12), the microbiota demonstrated higher activity in the functional pathways of lipoic acid metabolism, the bacterial secretion system, prodigiosin biosynthesis, and flagellar assembly (Figure 7B). Cross-sectional comparison at the cohort endpoint (CA12 vs. CF12) revealed that the microbiota in the caries-active children (CA12) was enriched in the functional pathways of Peptidoglycan biosynthesis and Aminoacyl-tRNA biosynthesis; in contrast, children who remained caries-free (CF12) showed greater activity in the pathways for Pentose and glucuronate interconversions and Thiamine metabolism (Figure 7C).

## 4. Discussion

The oral microbiota displays a complex composition and dynamic fluctuations within the salivary microenvironment. Disruption of the oral microbiome structure may lead to microecological disorders, contributing to several oral infectious diseases, including caries. Previous studies have elaborated on the existence of a core microbiota in health, such as *Streptococcus*, *Prevotella*, *Haemophilus*, and *Rothia* [9,24,25]. However, few studies have comprehensively demonstrated the role of interactions of salivary bacteria and fungi at the community level, both in the healthy status and caries development pathogenesis.

Oral fungi exhibit caries-related spatiotemporal distribution patterns in children [26,27]. In this study, salivary mycobiota profiling revealed that the predominant fungal taxa in both caries-free (CF) and caries-active (CA) children were constituted by commensal fungi and species that exist in the environment. The dominant fungal genera within the salivary microbiota included *Cladosporium*, *Malassezia*, *Alternaria*, *Candida*, and *Mortierella*. During caries development, besides the significantly changed salivary bacteria that were consistent with the changing trends reported in previous research, the salivary microbiota also exhibited various changes [6,28]. These results expand the current discovery of salivary mycobiota in children.

In addition, longitudinal analysis revealed several bacteria and fungi that were strongly associated with the dt index. Among the bacteria that presented a positive correlation with the dt index, *Desulfovibrio* sp. is a Gram-negative sulfate-reducing anaerobe, which converts sulfate to hydrogen sulfide via anaerobic respiration, and thus, may potentially damage epithelial cells. Previous studies found increased *Desulfovibrio* in orthodontic patients with fixed appliances, speculating its role in oral microbial shifts and metal corrosion [29]. Animal models implicated *Desulfovibrio* in canine periodontitis, though the species-specific functions still need further investigation [30].

Importantly, the fungus *Candida tropicalis* not only increases during caries development but also exhibits further elevation with increasing dt index, which indicates that it may play a role in the etiology and progression of dental caries. *Candida tropicalis* belongs to the *Candida* genus, which is a prevalent yeast species frequently isolated from both human and animal sources. This species is taxonomically closely related to *Candida albicans* and shares several pathogenic traits. It has emerged as an important pathogen associated with invasive candidiasis [31]. Biofilm formation is among the main virulence factors of *C. tropicalis*. These biofilms can become established and persist in different environments with a wide range of nutrients, pH, and osmolarity [32,33]. In addition, previous studies on salivary bacterial and fungal communities have suggested that the association between the Plaque Index (PLI) and fungal composition may stem from adhesive interactions between *Candida* and plaque bacteria, which could facilitate fungal retention. Concurrently, *Candida* promotes greater bacterial biomass accumulation [34]. *Candida* species thrive in carbohydrate-rich polymicrobial communities by utilizing glucose or lactate as carbon sources [35]. Levels of *Candida* and sugar-producing/acid-producing bacteria have been demonstrated to correlate positively [36]. This synergistic fungal–bacterial interaction collectively drives the initiation and progression of dental caries.

Multiple caries prediction models have been established [37,38]. Ahamd et al. [23] identified *Corynebacterium*, *Selenomonas, Kingella*, *Pseudomonas* sp., *Rhodotorula mucilaginosa*, and *Veillonella* parvula as potential early biomarkers of cariogenic transition by monitoring salivary microbiota from healthy, pre-carious, and caries-active states. Tseng et al. [33] developed a random forest model using plaque and saliva microbiota, identifying 20 bacterial taxa (including *S. mutans*, *Veillonella*, and *Prevotella*) for caries prediction, highlighting the complex community-level nature of caries development. These compositional shifts collectively implicate caries-associated microbial restructuring.

Bacteria *Brevundimonas diminuta* and *Bifidobacterium dentium* were both identified as critical contributors to caries-free status maintenance within the random forest modeling. Both species demonstrated significantly higher abundance in caries-free children than the children who developed caries. *Brevundimonas diminuta* is a Gram-negative aerobic opportunistic pathogen that causes infections in immunocompromised individuals and shows elevated abundance in the subgingival plaque of patients with severe periodontitis [39]. However, its relationship with dental caries has not been reported previously. *Bifidobacterium dentium* is an obligate anaerobe that ferments carbohydrates to produce acetic and lactic acids, and it is considered as an opportunistic cariogenic pathogen [40]. A previous study reported higher detection abundance of the *Bifidobacterium* genus in the saliva of children with caries [41]. This phenomenon may be associated with the metabolic characteristics of *Bifidobacterium*. It can metabolize saccharides to produce acid and exhibits strong acid tolerance, leading to a decrease in local oral pH. This subsequently triggers enamel demineralization and ultimately promotes the formation of carious lesions [42]. However, in this study, species-level analysis revealed that *B. dentium* exhibits features distinct from other *Bifidobacterium* species. The function of *B. dentium* in the salivary microecology of children with caries requires further exploration through biological investigation [41].

This study reveals two fungal species of *Malassezia* (*Malassezia restricta* and *Malassezia globosa*) play a significant role in maintaining a caries-free status in children. *Malassezia* spp. represents the most prevalent fungi colonizing on human skin from birth across all individuals. These fungi lack carbohydrate fermentation capacity and depend exclusively on lipid metabolism for growth [43]. Potential lipid sources in the oral cavity for *Malassezia* include saliva, gingival crevicular fluid, and host dietary lipids [43]. Furthermore, *Malassezia* demonstrates ecological associations with bacteria reliant on amino acid fermentation and exhibits a preference for slightly alkaline environments, reinforcing oral pH as a determinant factor for fungal community composition. Previous cross-sectional analyses of the salivary mycobiome in children with varying caries statuses revealed a higher relative abundance of *Candida*, *Cladosporium*, *Aspergillus*, and *Malassezia* in saliva, which have been consistently identified as core members of the human oral fungal mycobiota [44,45]. Studies based on dental plaque further indicated a stronger association of *Malassezia globosa* with caries-free status [5,46]. Moreover, analysis of the salivary fungal community in adults identified two clearly distinct community types at the genus level (mycotypes), with *Candida* and *Malassezia* being the primary fungi driving the clustering. It was also observed that acid-tolerant bacteria were enriched in the *Candida*-dominant mycotype, while pro-inflammatory bacteria increased in the *Malassezia*-dominant mycotype [47]. These findings suggest that *Malassezia*, as one of the key drivers of mycotype partitioning, need further investigation regarding its utility as a biomarker for oral diseases.

Notably, *Candida albicans* emerged as the sixth most significant fungus predictor for caries risk stratification. Previous studies indicate that *Candida* species can modulate caries risk by altering oral bacterial distribution [46,48]. *C. albicans* can interact with oral bacteria via physical attachment through fungal cell walls (e.g., surface proteins and extracellular polysaccharides, EPSs), extracellular signals, metabolite cross-feeding, and environmental changes [49,50]. In vitro studies show that *C. albicans* enhances biofilm virulence through synergistic symbiosis with *Streptococcus mutans*, co-metabolizing carbohydrates to accelerate enamel demineralization [51,52]. Research based on mother–child dyads also suggested the enrollment of *Candida* into the prevention and early diagnose of dental caries [53]. The consistent identification of this fungus across experimental paradigms underscores its potential etiological relevance in dental caries pathogenesis.

Longitudinal studies have shown distinct intra-microbial interaction networks in supragingival plaque between caries-active children and those remaining caries-free, indicating that bacterial interaction characteristics can serve as variables for caries risk stratification [22,27]. In this study, caries-active (CA12) children exhibited higher edge numbers and average connectivity in the total, intra-mycobial, and interkingdom bacterial–fungal networks, indicating active intra-mycobial and interkingdom interactions within the saliva microenvironment in the presence of caries. Conversely, intra-microbial network nodes and edges were fewer in CA12 group, suggesting reduced bacterial interaction activity in a caries-active state. However, it should be noted that these network characteristics, which were derived from sequencing data, still require validation by subsequent microbial experiments [51,54].

Notably, caries-susceptible children (the CA0 group, children were caries free at this moment as well as 12 months before caries detection) showed significantly lower bacterial alpha diversity but higher intra-microbial network edges and connectivity compared with caries-free (CF0/CF12) and caries-active (CA12) groups. This may indicate a transiently active bacterial state at the “pre-carious phase,” with the biological behavior and interaction dynamics of the microbiota at this time point requires further investigation.

Functional prediction based on 16S rRNA sequencing showed active phenylalanine, tyrosine, and tryptophan biosynthesis pathways in caries-free children. Tryptophan metabolism via the kynurenine pathway can suppress microbial growth by mediating host cell tryptophan metabolism to inhibit bacterial protein synthesis or energy acquisition [55]. This suggests host–microbe competition for tryptophan as a substrate, with distinct metabolic pathways generating bioactive metabolites that regulate host–microbiota interactions in cariogenesis [56].

In summary, this study elucidates the community-level bacterial–fungal interkingdom interactions and its mechanistic contributions to the promotion of cariogenic microenvironments. Compared with cross-sectional studies, the extending follow-up and increasing sampling time points facilitated the comprehensive observation of caries dynamics and long-term bacterial community and fungal community relationships. In addition, the strict inclusion/exclusion criteria ensured a reliable, homogeneous study population, thus effectively minimizing confounding variability and strengthening the reliability of the observed associations.

This study also has limitations. Participants were exclusively permanent residents of Beijing, China, with a relatively limited sample size; future validation should enroll larger cohorts from diverse geographical and socioeconomic backgrounds to enhance generalizability. The diagnosis of dental caries relied solely on visual inspection without radiographic assessment, which may have led to inaccuracies in caries detection, particularly in approximating surfaces. Moreover, as a multifactorial disease, caries involves more than microbiota; dietary habits and oral hygiene also influence incidence. Future studies should integrate lifestyle and dietary variables into caries risk models to improve accuracy, establishing a more realistic predictive system. In addition, given the single-center cohort limitation, external validation in multiethnic populations is required to establish diagnostic robustness before translating these microbial signatures into clinical caries-risk stratification tools.

## 5. Conclusions

This study established a prospective cohort to investigate the role of salivary bacterial community and fungal community interactions during the transition from caries-free to caries-active states, so as to characterize the bacterial and fungal features that contribute to cariogenic mechanisms. Specifically, salivary bacteria *Desulfovibrio*, *Bacteroides heparinolyticus*, *Alloprevotella*, *Anaerobiospirillum*, and *Candida tropicalis* not only showed altered abundances during caries development but also correlated with severity of caries, establishing diagnostic microbial signatures for caries prediction. This study further confirms the association of *Candida* and dental caries prediction, as well as the caries severity. Intraspecific interactions within the salivary mycobiota are likely to contribute to the cariogenic processes in preschool children.

## Figures and Tables

**Table 1:** Characteristics of network topological structure data.

Network Type	Group	Node	Edge	Average Network Connectivity	Average Path Length	Diameter
Total	CA0	394	1562	7.918934	1.966502	6.456498
	CA12	395	2116	10.71392	2.150628	11.34136
	CF0	420	1537	7.319048	4.250946	11.90128
	CF12	356	1080	6.067416	2.600149	8.836524
Bacteria to fungi	CA0	228	545	4.780702	2.410201	9.173526
	CA12	217	495	4.562212	1.69457	2
	CF0	225	408	3.626667	1.669015	5.684053
	CF12	176	288	3.272727	1.563037	2.570177
Bacteria	CA0	257	783	6.093385	3.820119	9.647465
	CA12	200	414	4.14	4.362419	12.15992
	CF0	278	551	3.964029	4.649041	14.1364
	CF12	259	468	3.6139	4.975864	12.69673
Fungi	CA0	149	464	6.228188	1.149706	4.021474
	CA12	173	910	10.52023	1.64026	5.713452
	CF0	121	274	4.528962	1.095462	4.001958
	CF12	131	426	6.5387	1.039188	2.425624

## Data Availability

The raw sequencing reads were deposited into the Genome Sequence Archive (GSA) database (Accession Number: CRA026916; CRA027046), (https://ngdc.cncb.ac.cn/gsub/submit/gsa/subCRA043776/finishedOverview, accessed on 17 June 2025; https://ngdc.cncb.ac.cn/gsub/submit/gsa/subCRA044056/finishedOverview, accessed on 20 June 2025).

## Supplementary Materials

The following supporting information can be downloaded at https://www.mdpi.com/article/10.3390/pathogens14101033/s1: Figure S1: Species accumulation curve. Table S1: General information on participants in the CF group. Table S2: General information on participants in the CA group. Table S3: Relative abundance of the bacteria at the species level in each group. Table S4: Relative abundance of the fungi at the species level in each group.
