# Supplementary material for: The Impact of Bacterial–Fungal Interactions on Childhood Caries Pathogenesis
Source: Pathogens. 2025 Oct 11;14(10):1033. doi: 10.3390/pathogens14101033 (PMC12567057; doi:10.3390/pathogens14101033)
Supplement: Supplementary file 1 [file pathogens-14-01033-s001.zip › pathogens-3888190-supplementary.pdf]

**Supplementary Materials**

## RESULTS

Participants in the cohort

Table S1: General information of participants in the CF group

| ID | Gender | Month of age | PLI index at baseline | PLI index at 12 months follow-up |
|---|---|---|---|---|
| CF1 | Male | 42 | 1 | 2 |
| CF2 | Male | 44 | 2 | 2 |
| CF3 | Female | 38 | 2 | 2 |
| CF4 | Female | 38 | 2 | 2 |
| CF5 | Female | 43 | 2 | 2 |
| CF6 | Female | 41 | 1 | 1 |
| CF7 | Female | 40 | 1 | 1 |
| CF8 | Female | 44 | 1 | 1 |
| CF9 | Female | 38 | 1 | 1 |
| CF10 | Female | 42 | 2 | 2 |
| CF11 | Male | 43 | 2 | 2 |
| CF12 | Male | 39 | 1 | 2 |
| CF13 | Male | 39 | 2 | 3 |
| CF14 | Female | 41 | 3 | 2 |
| CF15 | Male | 37 | 3 | 2 |
| CF16 | Female | 42 | 2 | 1 |
| CF17 | Male | 43 | 1 | 2 |

Mean PLI index at baseline: $1.65 \pm 0.52$.

Mean PLI index at 12 months follow-up: $1.82 \pm 0.39$.

Table S2. General information of participants in the CA group

| ID | Gender | Month of age | dt index | PLI index at baseline | PLI index at 12 months follow-up |
|---|---|---|---|---|---|
| CA1 | Female | 40 | 4 | 3 | 3 |
| CA2 | Male | 41 | 4 | 1 | 1 |
| CA3 | Female | 39 | 3 | 2 | 2 |
| CA4 | Female | 42 | 4 | 3 | 3 |
| CA5 | Male | 38 | 5 | 1 | 2 |
| CA6 | Male | 41 | 1 | 2 | 2 |
| CA7 | Male | 38 | 2 | 3 | 2 |
| CA8 | Female | 37 | 8 | 3 | 1 |
| CA9 | Female | 44 | 1 | 1 | 3 |
| CA10 | Male | 42 | 5 | 1 | 2 |
| CA11 | Male | 42 | 5 | 1 | 1 |
| CA12 | Male | 41 | 3 | 1 | 2 |
| CA13 | Female | 43 | 2 | 1 | 2 |
| CA14 | Male | 39 | 3 | 2 | 2 |
| CA15 | Female | 40 | 4 | 2 | 3 |
| CA16 | Female | 38 | 7 | 3 | 3 |
| CA17 | Male | 41 | 3 | 2 | 1 |
| CA18 | Male | 42 | 1 | 1 | 1 |
| CA19 | Female | 43 | 2 | 2 | 2 |
| CA20 | Female | 39 | 4 | 2 | 2 |
| CA21 | Female | 42 | 3 | 1 | 1 |

Mean PLI index at baseline: 1.86±0.71.

Mean PLI index at 12 months follow-up: 2.00±0.57.

## SPECIES ACCUMULATION CURVE

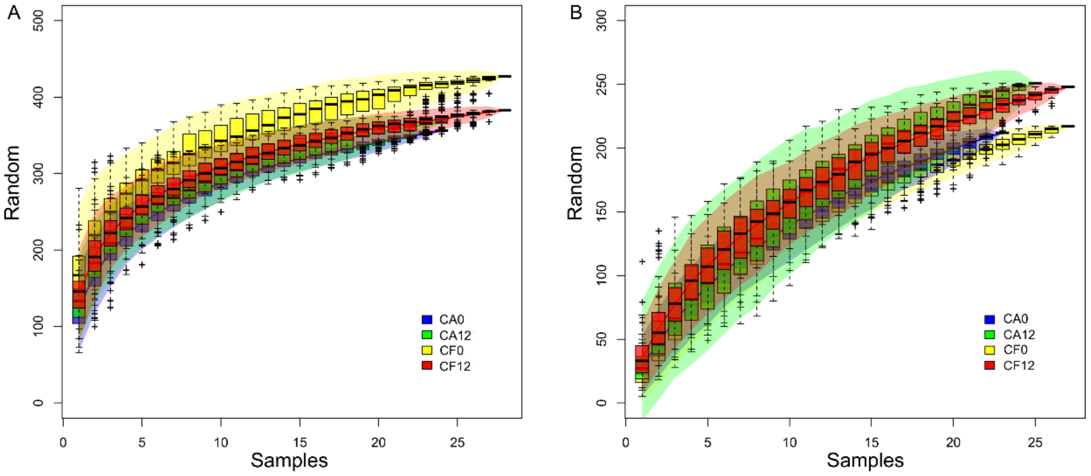


Figure S1: Species accumulation curve. (A) Bacteria, (B) Fungi. The abscissa of the species accumulation curve represents the sample size, and the ordinate represents the number of species. Each graph contains species accumulation curves for four groups of data at two time points, blue indicates CA0, green indicates CA12, yellow indicates CF0, and red indicates CF12.

**DOMINANT BACTERIAL SPECIES**

Table S3: Relative abundance of the bacteria at the species level in each group. Top 20 from 286 species are shown.

| Species | Relative abundance (%) | | | |
|---|---|---|---|---|
| | CF0 | CF12 | CA0 | CA12 |
| *Neisseria* | 10.64 | 20.49 | 12.92 | 15.70 |
| *Rothia mucilaginosa* | 3.79 | 11.06 | 15.54 | 10.71 |
| *Streptococcus* | 9.84 | 9.69 | 14.71 | 11.58 |
| *Streptococcus salivarius* | 3.77 | 5.17 | 7.18 | 8.03 |
| *Haemophilus parainfluenzae* | 4.02 | 7.93 | 6.03 | 6.93 |
| *Schaalia sp._HMT_180* | 8.34 | 4.77 | 4.77 | 4.93 |
| *Neisseria subflava* | - | 0.75 | 2.35 | 2.58 |
| *Prevotella melaninogenica* | 1.24 | 2.18 | 2.33 | 1.83 |
| *Veillonella dispar* | 2.09 | 2.94 | 1.91 | 1.66 |
| *Gemella* | 1.15 | 0.89 | 1.82 | 1.50 |
| *Granulicatella adiacens* | 1.42 | 1.26 | 1.69 | 1.24 |
| *Gemella sanguinis* | 1.12 | 0.58 | 1.63 | 0.94 |
| *Veillonella parvula* | 1.05 | 1.24 | 1.56 | 0.77 |
| *Porphyromonas pasteri* | 1.37 | 1.04 | 1.40 | 1.12 |
| *Veillonella atypica* | 1.27 | 1.60 | 1.39 | 2.22 |
| *Leptotrichia sp._HMT_417* | 1.08 | 1025 | 1.38 | 0.81 |
| *Lautropia mirabilis* | 2.61 | 1.83 | 1.18 | 1.66 |
| *Fusobacterium periodonticum* | 1.07 | 1.61 | 1.16 | 1.54 |
| *Streptococcus parasanguinis_clade_411* | 0.69 | 0.98 | 1.14 | 1.10 |
| *Actinomyces graevenitzii* | 1.62 | 3.14 | 1.04 | 1.40 |

Table S4: Relative abundance of the fungi at the species level in each group. Top 10 from 543 species are shown.

| Species | Relative abundance (%) | | | |
|---|---|---|---|---|
| | CF0 | CF12 | CA0 | CA12 |
| *Alternaria_angustiovoidea* | 10.47 | 17.19 | 10.97 | 18.24 |
| *Malassezia_globosa* | 6.54 | 8.64 | 15.81 | 11.77 |
| *Cladosporium_sp* | 10.99 | 13.41 | 3.14 | 9.70 |
| *Auricularia_auricula-judae* | - | - | - | 7.26 |
| *Cladosporium_herbarum* | 1.46 | 3.54 | 3.66 | 5.19 |
| *Monosporascus_sp* | 2.99 | 4.47 | 6.27 | 4.80 |
| *Malassezia_restricta* | 9.60 | 6.03 | 4.69 | 4.76 |
| *Cladosporium_tenuissimum* | 5.55 | 5.95 | 2.59 | 3.36 |
| *Candida_albicans* | 4.03 | 1.00 | 10.69 | 2.80 |
| *Nothophoma_macrospora* | 2.42 | 2.38 | 1.49 | 2.73 |
